# Nobiletin, as a Novel PDE4B Inhibitor, Alleviates Asthma Symptoms by Activating the cAMP-PKA-CREB Signaling Pathway

**DOI:** 10.3390/ijms251910406

**Published:** 2024-09-27

**Authors:** Yan Zhang, Yaping Yang, Huicong Liang, Yuerun Liang, Guixin Xiong, Fang Lu, Kan Yang, Qi Zou, Xiaomin Zhang, Guanhua Du, Ximing Xu, Jiejie Hao

**Affiliations:** 1 School of Medicine and Pharmacy, Ocean University of China, Qingdao 266003, China; zyola221199@163.com (Y.Z.); yaping0111@163.com (Y.Y.); lianghuicong@stu.ouc.edu.cn (H.L.); lyuerun@163.com (Y.L.); xgx980809@163.com (G.X.); lfangouc@163.com (F.L.); 13025114650@163.com (K.Y.); zouqi626513@163.com (Q.Z.); xiaominzhang91@163.com (X.Z.); 2 Marine Biomedical Research Institute of Qingdao, Qingdao 266071, China; yanz0122@163.com; 3 Qingdao Marine Science and Technology Center, Qingdao 266237, China

**Keywords:** asthma, cAMP-PKA-CREB signaling pathway, nobiletin, reverse docking, traditional chinese medicine

## Abstract

Asthma is a chronic airway inflammation that is considered a serious public health concern worldwide. Nobiletin (5,6,7,8,3′,4′-hexamethyl flavonoid), an important compound isolated from several traditional Chinese medicines, especially Citri Reticulatae Pericarpium, is widely used for a number of indications, including cancer, allergic diseases, and chronic inflammation. However, the mechanism by which nobiletin exerts its anti-asthmatic effect remains unclear. In this research, we comprehensively demonstrated the anti-asthmatic effects of nobiletin in an animal model of asthma. It was found that nobiletin significantly reduced the levels of inflammatory cells and cytokines in mice and alleviated airway hyperresponsiveness. To explore the target of nobiletin, we identified PDE4B as the target of nobiletin through pharmacophore modeling, molecular docking, molecular dynamics simulation, SPR, and enzyme activity assays. Subsequently, it was found that nobiletin could activate the cAMP-PKA-CREB signaling pathway downstream of PDE4B in mouse lung tissues. Additionally, we studied the anti-inflammatory and anti-airway remodeling effects of nobiletin in LPS-induced RAW264.7 cells and TGF-β1-induced ASM cells, confirming the activation of the cAMP-PKA-CREB signaling pathway by nobiletin. Further validation in PDE4B-deficient RAW264.7 cells confirmed that the increase in cAMP levels induced by nobiletin depended on the inhibition of PDE4B. In conclusion, nobiletin exerts anti-asthmatic activity by targeting PDE4B and activating the cAMP-PKA-CREB signaling pathway.

## 1. Introduction

Asthma is a chronic bronchial inflammation disorder in which a variety of cells and cellular components play important roles. In recent decades, the prevalence of asthma has increased dramatically around the world, with reported populations of up to 300 million [1,2]. Currently, the main therapeutic drugs for asthma are glucocorticoids, theophylline, β-receptor agonists, anticholinergics, and lipoxygenase inhibitors [3]. However, these agents are also associated with adverse reactions. Therefore, a detailed understanding of the mechanisms and the discovery of novel candidate compounds may prove to be significantly invaluable for the treatment of asthma.

Chronic inflammation in asthma is associated with airway hyperresponsiveness, typically resulting in reversible expiratory airflow restriction [4]. It is characterized by a variety of clinical and pathological features, including airway eosinophilia, increased mucus production, airway remodeling, and bronchoconstriction. Th2 cytokines play an important role in the pathogenesis of asthma [5]. Of these cytokines, IL-13 induces the transition of airway epithelial cells into mucus-secreting phenotypes and promotes goblet cell maturation, mucus secretion, and airway hyperresponsiveness. Meanwhile, IL-4 drives IgE class switching and, in conjunction with IL-13, is required to produce high-affinity IgE [6,7]. IgE mediates mast cell and basophil degranulation by crosslinking FcεRI upon allergen recognition. When activated, mast cells cause the immediate release of histamine, protease, and other inflammatory mediators, leading to smooth muscle contraction in the airways [8,9,10,11,12]. Additionally, IL-5 can promote the growth, survival, differentiation, and recruitment of eosinophils [13], which are the main inflammatory cells that cause asthma, and release mediators that induce bronchoconstriction [14]. During the progression of asthma, another important cytokine, transforming growth factor β1 (TGF-β1), constantly stimulates airway smooth muscle (ASM) cells and lung fibroblasts, leading to smooth muscle cell hyperplasia and sub-epithelial fibrosis, respectively. These are the characteristic features of airway remodeling [15,16]. This results in persistent airflow obstruction [17,18]. Based on research, certain methods that could suppress airway inflammation or remodeling are regarded as potential strategies to treat asthma. Phosphodiesterase 4 (PDE4) is well known to be closely associated with various key asthma pathological processes, as described above. Therefore, targeting PDE4 has been proposed as a promising approach for treating patients with asthma [19].

Early preclinical studies in murine models of asthma have documented the ability of PDE4 inhibitors to inhibit the recruitment of eosinophils and bronchial hyperresponsiveness [19,20]. PDE4 selectively degrades 3′,5′-cyclic adenosine monophosphate (cAMP), which is a well-known second messenger that has been shown to contribute to anti-inflammatory activity and interfere with remodeling and relaxation of ASM cells [21]. Previous studies have shown that PDE inhibitors could elevate cAMP levels, resulting in the inhibition of airway inflammation and remodeling [22,23,24]. PDE4 subtypes are expressed in immune and inflammatory cells at diverse levels, with PDE4A being expressed at low levels in inflammatory cells, and PDE4D being expressed in the area postrema/nucleus of the solitary tract. Conversely, PDE4B is expressed in inflammatory cells and ASM cells [25,26]. The upregulation of cAMP by PDE4 inhibitors has been widely studied in many diseases, such as asthma, COPD, and psoriasis [27]. Apremilast is a small-molecule PDE4 inhibitor that was approved by the US Food and Drug Administration in 2014 for the treatment of psoriasis and psoriatic arthritis. Clinical trials have shown that it is well tolerated and causes few adverse reactions [28]. Protein kinase A (PKA) is one of the important classical downstream signals of cAMP. Upon activation of PKA, the regulatory subunit is altered to release the catalytic subunit (PKA-C), which then increases the phosphorylation of the cAMP response element-binding protein (CREB). In addition, PKA activation can suppress nuclear factor kappa-B (NF-κB) activity through indirect competition for the transcriptional coactivator CREB binding protein or its homologous protein p300, or through modification of the C-terminal transactivation domain of the NF-κB subunit p65 [29,30,31]. Some studies have shown that ovalbumin (OVA) can activate NF-κB, leading to the increased expression of inflammatory factors and intensification of Th2-related inflammatory responses. Therefore, inhibition of NF-κB by natural products, functional peptides, and traditional Chinese medicine has been reported to improve the symptoms of OVA-induced allergic asthma [32,33,34,35,36,37].

**Figure 1:**
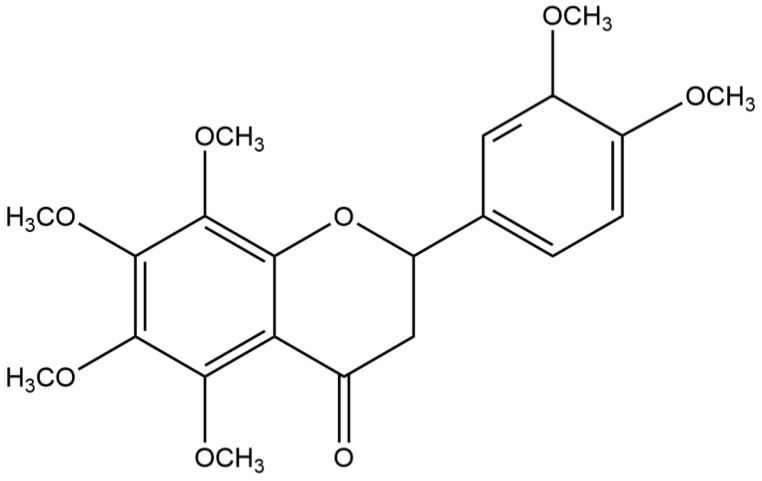
Chemical structure of nobiletin.

Recently, there has been increasing attention paid to the treatment of asthma using traditional Chinese medicine. This treatment has been proven effective in clinical trials, such as Mai-men-dong-tang and Dingchuan-tang, for patients with asthma in China and other countries [32,38,39,40,41]. Nobiletin (Figure 1) is one of the most well-studied compounds in polymethoxyflavonoids, and it is mainly isolated from the peel of citrus fruits. Nobiletin has been proven to be a multi-functional pharmaceutical agent beneficial to various human systems, with anti-cancer, anti-inflammatory, anti-oxidant, and other bioactivities [42,43]. Meanwhile, studies have also shown that nobiletin can suppress airway eosinophilic inflammation in asthmatic rats and inhibit ASM cell contraction [44,45,46]. These activities indicate that nobiletin has some anti-asthmatic effects. However, the mechanism by which nobiletin alleviates asthma symptoms has not been fully elucidated. Therefore, the present study has been undertaken to evaluate the therapeutic effects of nobiletin on asthma using an OVA-induced mouse asthma model, a RAW264.7 inflammation model, and an ASM proliferation model, and to further investigate its potential mechanism of action. Specifically, the inhibitory effect of nobiletin on PDE4B and the relief of asthma symptoms via activation of the downstream cAMP-PKA-CREB signaling pathway were investigated in depth. All of these studies are expected to lay the foundation for the secondary development of traditional Chinese medicine.

## 2. Results

### 2.1. Nobiletin Decreased the Levels of Inflammation in Bronchoalveolar Lavage Fluid (BALF) and the Content of Specific IgE in Serum

**Figure 2:**
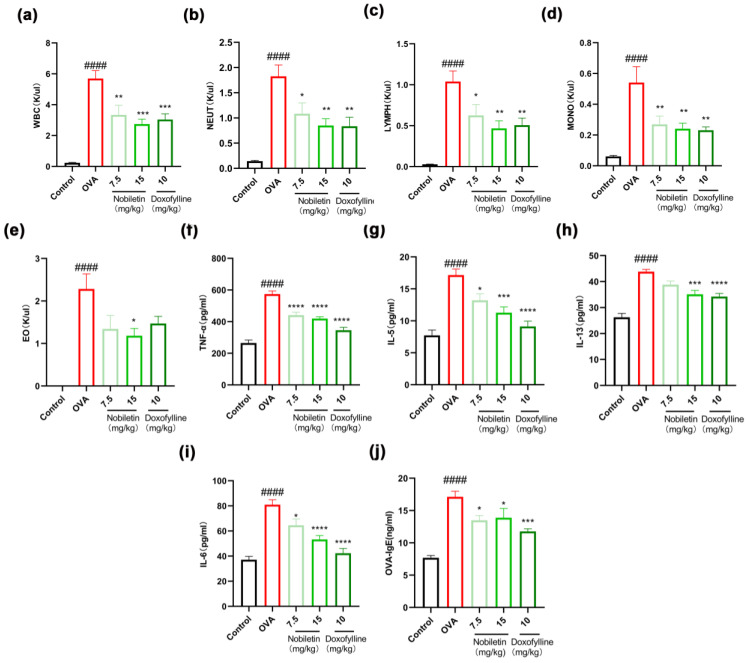
Nobiletin decreased the levels of inflammation in the BALF and the content of OVA-IgE in the serum. (**a**–**e**) Total inflammatory cells, neutrophils, lymphocytes, monocytes, and eosinophils in the BALF. (**f**) TNF-α levels in the BALF. (**g**) IL-5 levels in the BALF. (**h**) IL-13 levels in the BALF. (**i**) IL-6 levels in the BALF. (**j**) OVA-IgE levels in the serum. Values represent the mean ± SEM. (**a**–**i**, *n* = 8; **j**, *n* = 6). * *p* < 0.05, ** *p* < 0.01, *** *p* < 0.001, **** *p* < 0.0001 vs. the OVA group. #### *p* < 0.0001 vs. the control group.

In the development of asthma, the appearance of inflammation appears to be crucial. In this experiment, we tested the effects of nobiletin on inflammatory cells. Ovalbumin (OVA) can induce asthmatic responses in mice. As shown in Figure 2a–e, compared to the control group, the number of white blood cells, neutrophils, lymphocytes, monocytes, and eosinophils in the BALF of mice in the OVA group increased markedly. Nobiletin reduced the number of inflammatory cells in asthmatic mice. The levels of TNF-α, IL-5, IL-13, and IL-6 in the BALF were detected by enzyme-linked immunosorbent assay (ELISA), and the same methods were used to detect OVA-IgE levels in the serum, as shown in Figure 2f–j. The levels of TNF-α, IL-5, IL-13, and IL-6 in the BALF supernatant and the OVA-IgE content in the serum increased markedly in the OVA group, while the levels of inflammatory factors improved to different degrees after nobiletin treatment. The results showed that treatment with nobiletin resulted in a significant decrease in the levels of inflammation.

### 2.2. Nobiletin Attenuated Enhanced Pause (Penh) in Asthmatic Mice

**Figure 3:**
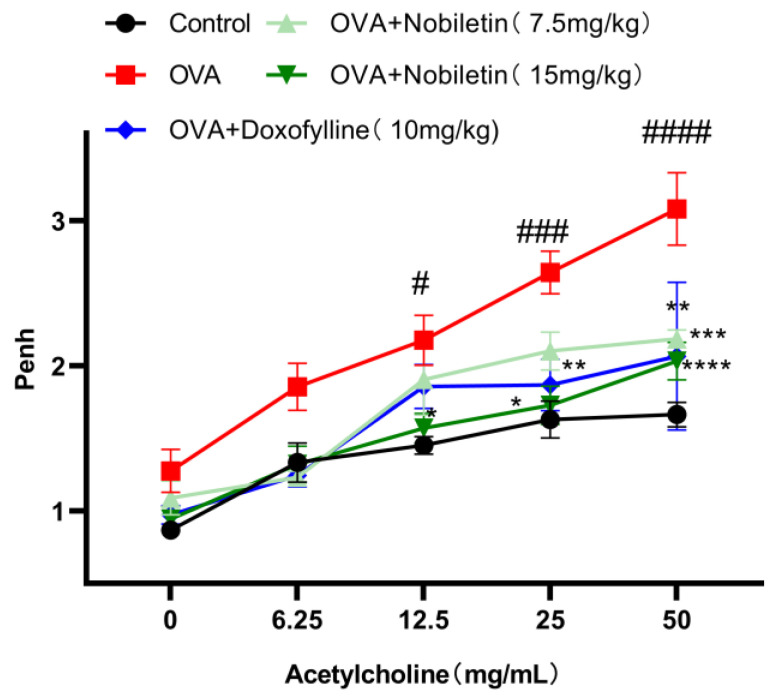
Nobiletin decreased the Penh in asthmatic mice. 0, 6.25, 12.5, 25, and 50 mg/mL of ACh were added for atomization. Values represent the mean ± SEM. (*n* = 4). * *p* < 0.05, ** *p* < 0.01, *** *p* < 0.001, **** *p* < 0.0001 vs. the OVA group. # *p* < 0.05, ### *p* < 0.001, #### *p* < 0.0001 vs. the control group.

Airway hyperresponsiveness refers to excessive airway constriction and is one of the most obvious features of asthma. Within 24 h after the last atomization, mice were placed on a non-invasive airway detection instrument. After stabilization of breathing, 0, 6.25, 12.5, 25, and 50 mg/mL of acetylcholine (ACh) were added for atomization to evaluate the airway hyperresponsiveness of the mice. As expected, compared to the control group, Penh increased after high concentrations of ACh atomization in the OVA group, while the airway hyperresponsiveness of the mice was reduced after treatment with nobiletin (Figure 3).

### 2.3. Effect of Nobiletin on Pathological Changes in Lung Tissue in Asthmatic Mice

**Figure 4:**
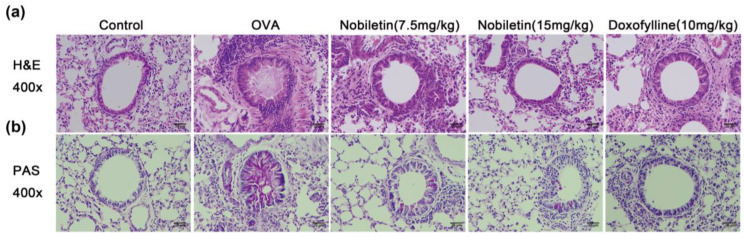
Effect of nobiletin on pathological changes in lung tissue in asthmatic mice. (**a**) H&E staining was used to detect inflammatory cell infiltration. (**b**) PAS staining was used to detect the production of mucus around the airways. 400× magnification; scale bar: 20 μm (*n* = 4).

One of the main characteristics of asthmatic lungs is the infiltration of inflammatory cells and secretion of mucus. Hematoxylin and eosin (H&E) staining was used to detect the degree of inflammatory cell infiltration in lung tissue, while periodic acid-Schiff (PAS) staining was used to detect mucus secretion in lung tissue. As shown in Figure 4, H&E staining revealed that the OVA group had marked inflammatory cell infiltration in the bronchial lumen compared to the control group. Furthermore, PAS staining showed that there was significant mucus secretion and many goblet cells in the lung tissue of mice in the OVA group. The infiltration of inflammatory cells and mucus secretion in mice was improved after nobiletin treatment.

### 2.4. Molecular Docking and Molecular Dynamics (MD) between Nobiletin and PDE4B

**Figure 5:**
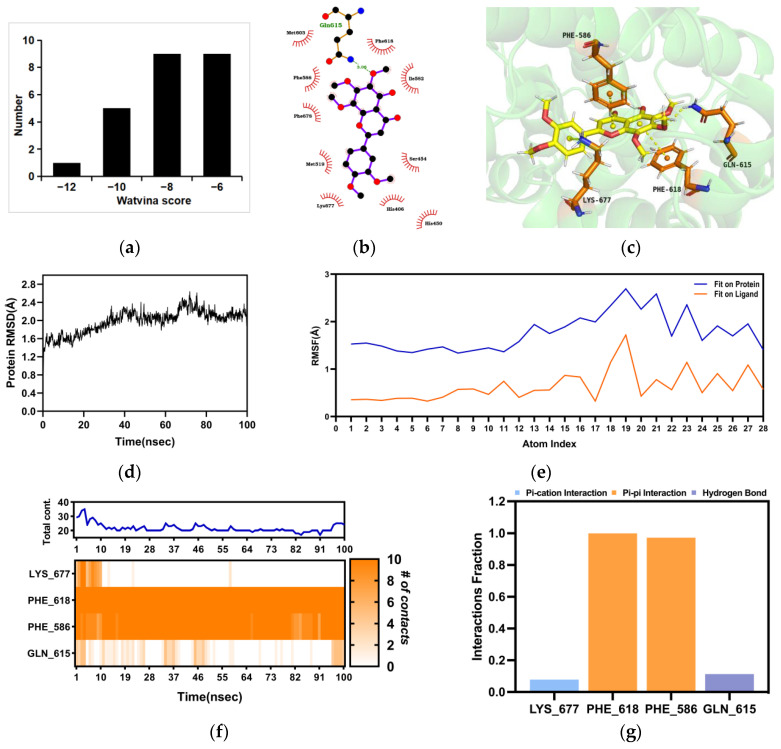
Interaction between PDE4B and nobiletin. (**a**) The docking score distribution diagram of nobiletin and 24 crystal structures of PDE4B. (**b**) The 2D interaction structure between nobiletin and PDE4B. Hydrogen bonds are shown as green dotted lines, while the spoked arcs represent residues making nonbonded contacts with the ligand. (**c**) The 3D interaction structure between nobiletin and PDE4B. (**d**) The Root Mean Square Deviation (RMSD) of PDE4B’s backbone. (**e**) The Ligand Root Mean Square Fluctuation (L-RMSF) of nobiletin in PDE4B. (**f**) The interactions between key residues and nobiletin during the entire simulation process. (**g**) The main fraction of interactions between nobiletin and PDE4B.

The anti-asthmatic activity of nobiletin has been elucidated. In order to reveal its targets, we used the Discovery Studio method to perform reverse target fishing. Nobiletin was virtually matched with the pharmacophore models individually, and it was found that PDE4B scored the highest. In addition, molecular docking was performed to study the interactions between nobiletin and PDE4B. 24 crystal structures of PDE4B were obtained from the Protein Data Bank (PDB), and the docking score of these 24 crystal structures and nobiletin was calculated by Watvina. The docking score distribution of nobiletin and the 24 crystal structures of PDE4B are shown in Figure 5a. PDE4B (PDBID:4myq) had the best score with nobiletin, with a score of −12.29. The docking results are shown in Figure 5b,c. The binding stability of nobiletin and PDE4B (PDBID:4myq) was further simulated by MD simulations. The Root Mean Square Deviation (RMSD) of the PDE4B backbone in the complex was <2.8 Å, indicating that PDE4B had a stable conformation during the simulation (Figure 5d). The Root Mean Square Fluctuation (RMSF) values of atoms 19, 21, and 23 fluctuated greatly relative to the protein, while the other atoms showed relatively stable atomic fluctuations, indicating a relatively firm binding to the protein pocket (Figure 5e). As shown in Figure 5f,g, GLN 615, PHE 618, PHE 586, and LYS 677 were key residues. The pi-pi interaction fraction values corresponding to PHE 618 and PHE 586 were both around 1.0, which were 0.97 and 1.11, respectively, indicating that these two residues maintained constant interactions throughout the simulation time.

### 2.5. The Binding of the PDE4B Protein with Nobiletin

**Figure 6:**
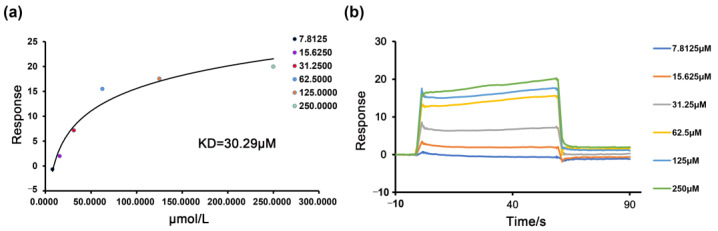
The binding force between PDE4B and nobiletin was detected by SPR. (**a**) Affinity model diagram of nobiletin and PDE4B. (**b**) Kinetic binding diagram of nobiletin and PDE4B.

To validate the interaction of nobiletin with the PDE4B protein, the surface plasmon resonance assay (SPR) method was used to evaluate the binding capacity between nobiletin and the PDE4B protein. We observed that nobiletin interacted with the PDE4B protein with a KD (affinity constant) of 30.29 µmol/L (Figure 6). The value of the affinity constant KD can directly reflect the binding ability between small molecules and proteins. Our results proved that the PDE4B protein and nobiletin had a strong binding ability.

### 2.6. The Inhibitory Effect of Nobiletin on PDE4B

**Figure 7:**
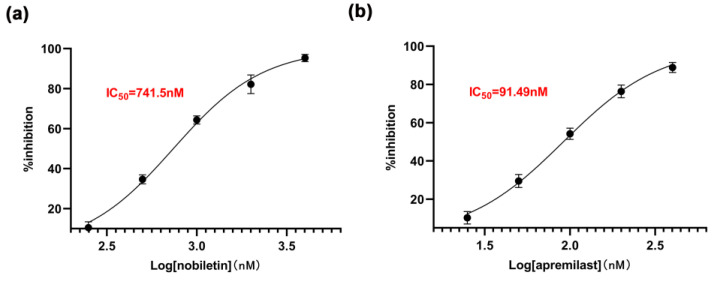
The inhibitory effect of nobiletin on PDE4B. (**a**) The inhibition rate of PDE4B by nobiletin at different concentrations. (**b**) The inhibition rate of PDE4B by apremilast at different concentrations. The values represent the mean ± SEM (*n* = 3).

To directly demonstrate the inhibitory effect of nobiletin on PDE4B, the inhibition rates of nobiletin at concentrations of 0.25 to 4 μM and apremilast at concentrations of 25 to 400 nM were determined individually. The IC_50_ values were then calculated. As shown in Figure 7, the IC_50_ of nobiletin for inhibiting the PDE4B enzyme is 741.5 nM, while that of apremilast is 91.49 nM. These results confirm that nobiletin can inhibit the activity of the PDE4B enzyme.

### 2.7. Effect of Nobiletin on the cAMP-PKA-CREB and NF-κB Signaling Pathways

**Figure 8:**
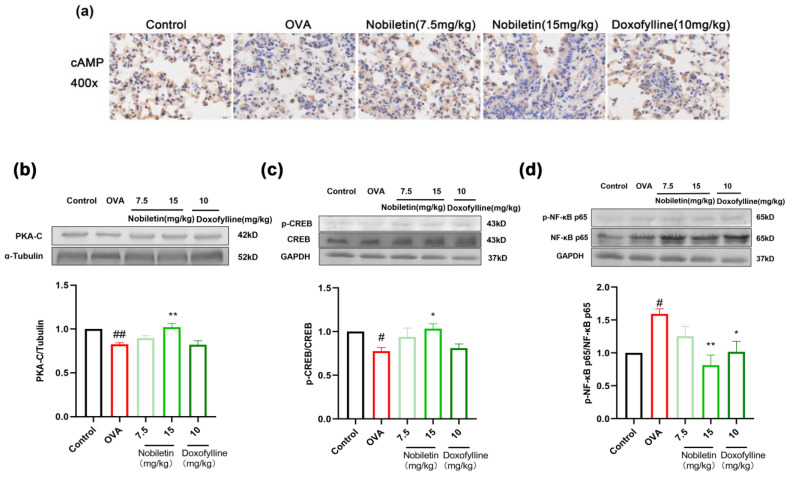
Effect of nobiletin on the cAMP-PKA-CREB and NF-κB signaling pathways. (**a**) The expression of cAMP was investigated by immunohistochemical staining. (**b**) Western blot analysis of PKA-C, α-tubulin, and quantification of the proteins. (**c**) Western blot analysis of p-CREB, CREB, and GAPDH and quantification of the proteins. (**d**) Western blot analysis of p-NF-κB p65, NF-κB p65, GAPDH, and quantification of the proteins. Values represent the mean ± SEM. (*n* = 5). * *p* < 0.05, ** *p* < 0.01 vs. the OVA group. # *p* < 0.05, ## *p* < 0.01 vs. the control group. 400× magnification; scale bar: 20 μm.

The cAMP-PKA-CREB signaling pathway downstream of PDE4B was explored in mouse lung tissues to verify that PDE4B is the anti-asthmatic target of nobiletin. The cAMP content in mouse lung tissue was detected by immunohistochemistry, and the levels of PKA-C, p-CREB, and p-NF-κB were detected by Western blotting. As shown in Figure 8a, the expression of cAMP was lower in the OVA group than in the control group and increased with 7.5 and 15 mg/kg of nobiletin. Compared to the control group, the levels of PKA-C and p-CREB proteins decreased in the lung tissue of the OVA group, and the phosphorylation of NF-κB was increased. After treatment with nobiletin, the levels of PKA-C and p-CREB increased, whereas p-NF-κB levels decreased (Figure 8b–d). These results indicated that the anti-asthma effects of nobiletin may be mediated by the cAMP-PKA-CREB and NF-κB signaling pathways.

### 2.8. Nobiletin Decreased the Level of Inflammation in RAW264.7 Cells

**Figure 9:**
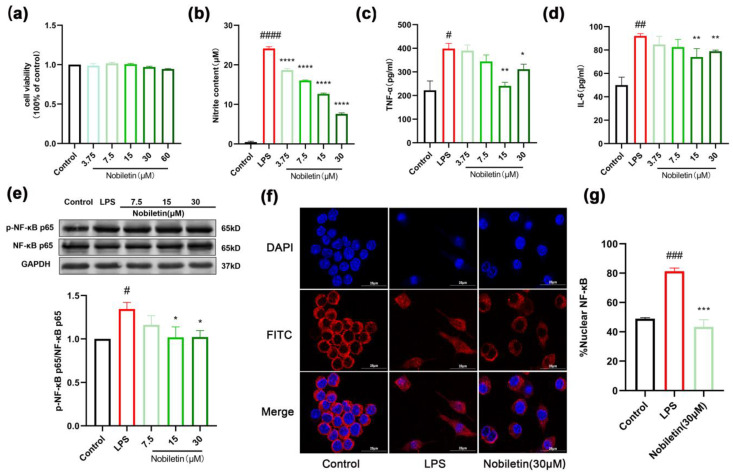
Nobiletin decreased the level of LPS-induced inflammation in RAW264.7 cells. RAW264.7 cells were treated with 1 μg/L of LPS and increasing concentrations (3.75, 7.5, 15, 30, 60 μM) of nobiletin for 24 h. (**a**) Cell viability after treatment with various concentrations of nobiletin for 24 h. (**b**) Nitrite content after LPS and nobiletin treatment. (**c**) TNF-α levels after LPS and nobiletin treatment. (**d**) IL-6 levels after LPS and nobiletin treatment. (**e**) Western blot analysis of p-NF-κB p65, NF-κB p65, and GAPDH and quantification of the proteins. (**f**) Effects of LPS and nobiletin treatment on NF-κB signaling by immunofluorescence staining. Scale bar: 25 μm. (**g**) Quantification of NF-κB p65 nuclear distribution in these images is represented. Values represent the mean ± SEM. (**a**–**e**, *n* = 6; **f**,**g**, *n* = 3). * *p* < 0.05, ** *p* < 0.01, *** *p* < 0.001, **** *p* < 0.0001 vs. the LPS group; # *p* < 0.05, ## *p* < 0.01, ### *p* < 0.001, #### *p* < 0.0001 vs. the control group.

In this study, the anti-inflammatory activity of nobiletin in RAW264.7 cells was further investigated based on its anti-asthmatic effect in vivo. The results of the MTT assay showed that nobiletin had no obvious toxic effect on cells at concentrations below 60 μM (Figure 9a). Concentrations of 3.75, 7.5, 15, and 30 μM were selected for follow-up experiments. The cells were incubated with 1 μg/mL of lipopolysaccharide (LPS) for 24 h to detect the level of inflammation. The results indicated that compared to the LPS group, the levels of nitrite content, TNF-α, and IL-6 in the cells were markedly reduced by nobiletin (Figure 9b–d). To determine the effects of nobiletin on the inflammatory signaling pathway, we detected the expression of p-NF-κB stimulated by LPS in RAW264.7 cells. The content of p-NF-κB increased in the LPS group, while nobiletin at different concentrations markedly inhibited NF-κB phosphorylation (Figure 9e). Furthermore, we also detected the nuclear transcription of NF-κB protein through immunofluorescence. In the control group, NF-κB was primarily concentrated in the cytoplasm, while LPS stimulation increased the content of NF-κB in the nucleus. Nobiletin significantly inhibited the nuclear transport of NF-κB protein (*p* < 0.001, Figure 9f,g). These results indicated that nobiletin has significant anti-inflammatory activity in LPS-stimulated RAW264.7 cells.

### 2.9. Effects of Nobiletin on the cAMP-PKA-CREB Signaling Pathway in RAW264.7 Cells

**Figure 10:**
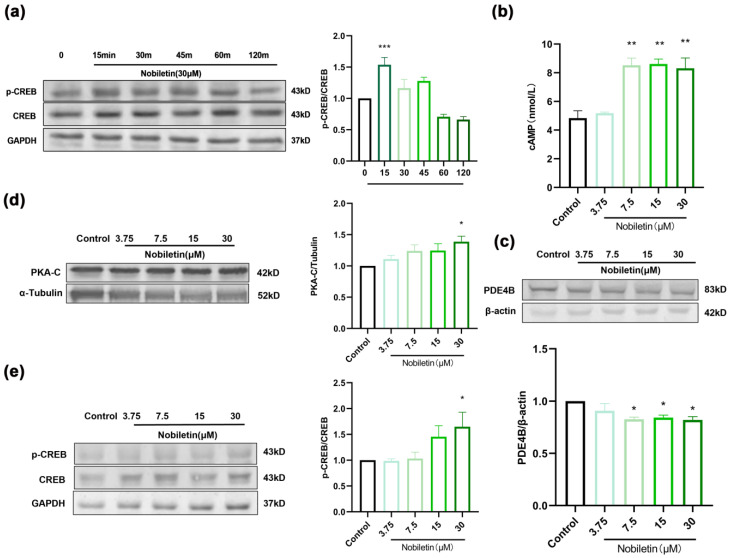
Effects of nobiletin on the cAMP-PKA-CREB signaling pathway in RAW264.7 cells. RAW264.7 cells were treated with various concentrations (3.75, 7.5, 15, and 30 µM) of nobiletin for 15 min. (**a**) The effect of time on p-CREB protein was analyzed by Western blotting. (**b**) cAMP levels after treatment with various concentrations of nobiletin for 15 min. (**c**) Western blot analysis of PDE4B, β-actin, and quantification of the proteins. (**d**) Western blot analysis of PKA-C, α-Tubulin, and protein quantification. (**e**) Western blot analysis of p-CREB, CREB, and GAPDH, and quantification of the proteins. Values represent the mean ± SEM. (**a**,**c**, *n* = 4; **b**,**d**,**e**, *n* = 6). * *p* < 0.05, ** *p* < 0.01, *** *p* < 0.001 vs. the control group.

To identify the signaling pathways that mediate the effects of nobiletin in RAW264.7 cells, 30 μM nobiletin was added and allowed to incubate for 15, 30, 45, 60, and 120 min. The results showed that CREB phosphorylation was highest after 15 min of incubation (Figure 10a). Therefore, nobiletin stimulation for 15 min was selected for the follow-up experiments. After incubation with different concentrations of nobiletin for 15 min, intracellular cAMP levels were detected by ELISA. Compared with the control group, nobiletin significantly increased cAMP levels (** *p* <0.01, Figure 10b). The levels of PDE4B, PKA-C, and p-CREB in cells were detected by Western blotting. Thus, PDE4B levels were reduced and the levels of PKA-C and p-CREB were increased by nobiletin (Figure 10c–e). These results suggest that nobiletin plays an anti-inflammatory role through the cAMP-PKA-CREB signaling pathway. The targets and signaling pathways of nobiletin were verified.

### 2.10. Nobiletin Decreased the Proliferation of ASM Cells Induced by TGF-β1

**Figure 11:**
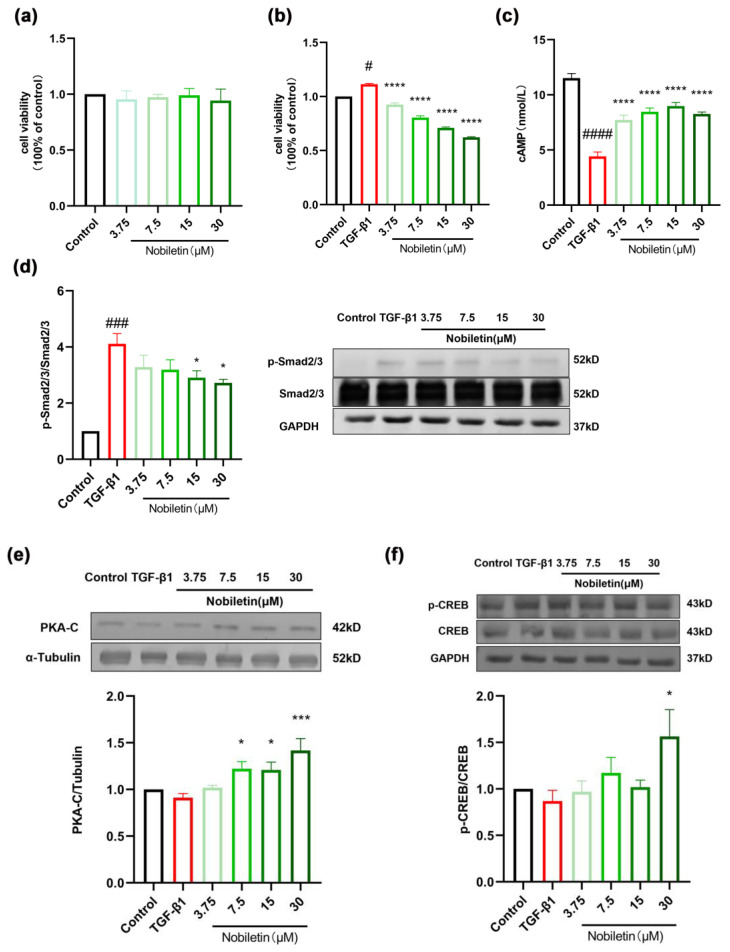

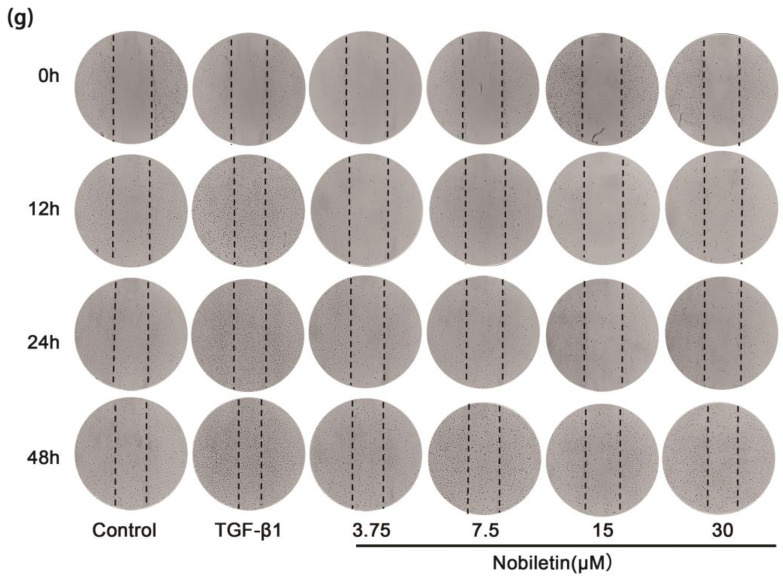
Nobiletin inhibits ASM cell proliferation and the TGF-β1 signaling pathway. (**a**) Cell viability after treatment with various concentrations of nobiletin for 24 h. (**b**) Cell viability after treatment with TGF-β1 and nobiletin for 24 h. (**c**) cAMP levels after treatment with TGF-β1 and nobiletin for 24 h. (**d**) Western blot analysis of p-Smad2/3, Smad2/3, and GAPDH, and quantification of the proteins. (**e**) Western blot analysis of PKA-C, α-Tubulin, and quantification of the proteins. (**f**) Western blot analysis of p-CREB, CREB, and GAPDH, and quantification of the proteins. (**g**) Cell proliferation and degree of migration after treatment with TGF-β1 and nobiletin for 0, 12, 24, and 48 h using the scratch assay. Values represent the mean ± SEM. (**a**–**f**, *n* = 6; **g**, *n* = 3). * *p* < 0.05,*** *p* < 0.001, **** *p* < 0.0001 vs. the TGF-β1 group. # *p* < 0.05, ### *p* < 0.001, #### *p* < 0.0001 vs. the control group.

Based on these results, we investigated the anti-proliferative effect of nobiletin on ASM cells. The results of the MTT assay showed that nobiletin had no obvious cytotoxicity in ASM cells at concentrations of up to 30 µM (Figure 11a). Therefore, concentrations of 3.75, 7.5, 15, and 30 µM were selected for subsequent experiments. After 12 h of starvation, ASM cells were incubated with 10 ng/mL of TGF-β1 and nobiletin for 24 h. Compared to the control group, TGF-β1 significantly induced cell proliferation, while nobiletin treatment could reduce cell proliferation (**** *p* <0.0001, Figure 11b). Additionally, we simulated cell proliferation and migration in vivo through an in vitro scratch assay. Compared to the control group, cells exposed to TGF-β1 migrated to the central position after 12, 24, and 48 h, especially at 48 h, and the number of cell migrations was remarkably reduced after treatment with nobiletin (Figure 11g). The results showed that nobiletin could reduce the migration of ASM cells induced by TGF-β1. Western blotting was used to detect the content of p-Smad2/3 in cells. As shown in Figure 11d, TGF-β1 stimulation could induce Smad2/3 phosphorylation, while nobiletin treatment reduced the level of intracellular p-Smad2/3. These results suggested that nobiletin plays a role in anti-airway remodeling through the TGF-β1/Smad signaling pathway.

### 2.11. Effects of Nobiletin on the cAMP-PKA-CREB Signaling Pathway in ASM Cells Induced by TGF-β1

Our studies on lung tissues and RAW264.7 cells have shown that nobiletin activates the cAMP-PKA-CREB signaling pathway downstream of PDE4B. In order to further ascertain the mechanism of action of nobiletin, we conducted investigations on ASM cells. The results of the ELISA assay demonstrated that compared to the control group, the TGF-β1 group significantly reduced the levels of cAMP, while treatment with nobiletin reversed the decrease in cAMP levels induced by the TGF-β1 group (**** *p* <0.0001, Figure 11c). Western blotting was used to detect the protein levels of PKA-C and p-CREB. As shown in Figure 11e,f, nobiletin increased the content of PKA-C and p-CREB. These results demonstrated that nobiletin could activate the cAMP-PKA-CREB signaling pathway.

### 2.12. Nobiletin Played a Role in Increasing cAMP Levels by Depending on PDE4B

**Figure 12:**
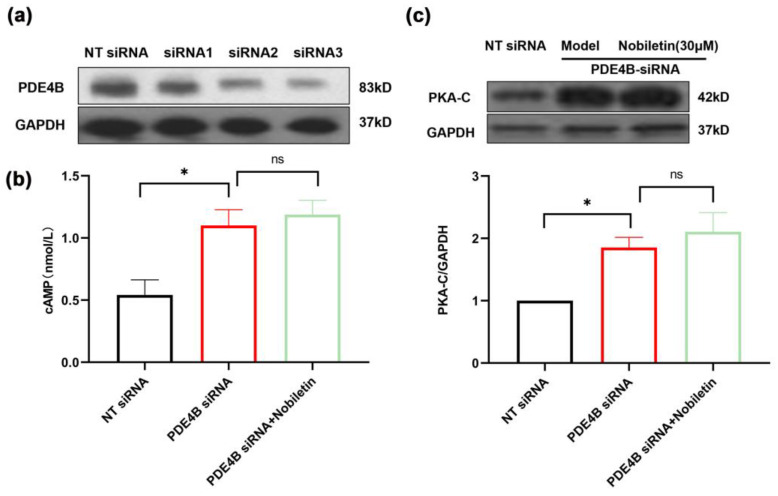
Nobiletin depends on PDE4B to increase cAMP and PKA-C levels in RAW264.7 cells. (**a**) Western blot analysis of PDE4B in three PDE4B siRNA-transfected RAW264.7 cells. (**b**) cAMP levels after treatment with nobiletin in PDE4B siRNA-transfected RAW264.7 cells. (**c**) Western blot analysis of PKA-C and GAPDH, and quantification of proteins in PDE4B siRNA-transfected RAW264.7 cells. Values represent the mean ± SEM (*n* = 3). ns represented *p* > 0.05; * *p* < 0.05 vs. the NT siRNA group.

We added reverse validation experiments to better establish that nobiletin acts through PDE4B, based on the results showing that PDE4B is the target of nobiletin. Three different siRNAs targeting PDE4B (siRNA1, siRNA2, and siRNA3) were transfected into RAW264.7 cells, and siRNA3 was selected for use in the present study (Figure 12a). We investigated the modulation of cAMP and PKA-C levels by nobiletin in cells transfected with PDE4B siRNA. It was found that cAMP and PKA-C levels did not significantly increase in the presence of nobiletin treatment compared to the group without nobiletin (Figure 12b,c). These results suggested that nobiletin increases cAMP levels by depending on PDE4B, thereby playing a role in the treatment of asthma.

## 3. Discussion

**Figure 13:**
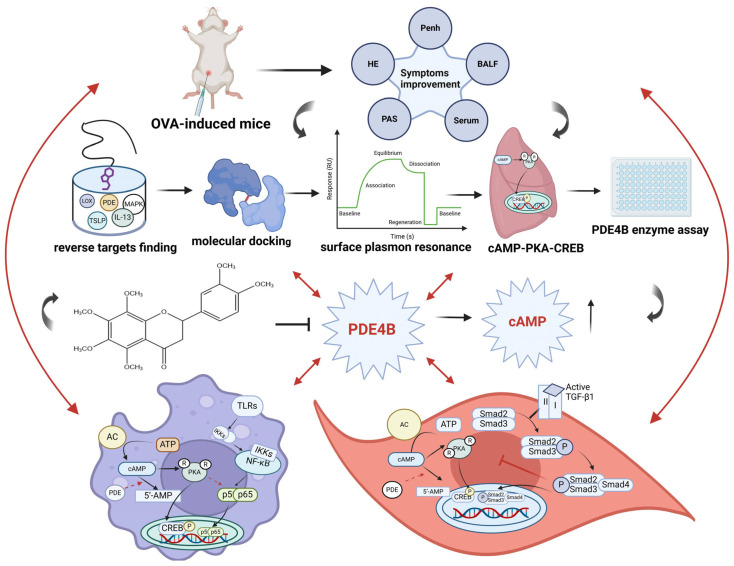
The proposed activity and mechanisms of the anti-asthma effect of nobiletin (images were created on BioRender.com).

Nobiletin, a type of polymethoxyflavone compound, can exert its effects through multiple targets in a synergistic manner. In addition, studies have shown that an increased number of methoxy groups in polymethoxyflavones can enhance the permeability of biological membranes, leading to increased cellular uptake and improved bioactivity compared to non-polymethoxyflavones [47]. Many flavonoid compounds, including eupatilin, have demonstrated anti-asthmatic activity. Our research has found that nobiletin also exhibits good anti-asthmatic effects at lower doses and is more effective than eupatilin [48]. Furthermore, studies have shown that nobiletin has a stronger anti-inflammatory effect on LPS-induced RAW264.7 cells compared to other polymethoxyflavone compounds [49]. This confirms the superior efficacy of nobiletin. Additionally, studies have shown that the metabolites of nobiletin may be more effective than the parent compound, suggesting the potential of nobiletin as a prodrug [50]. In the present study, we comprehensively investigated the anti-asthmatic activity of nobiletin. First, we demonstrated that nobiletin treatment could markedly improve asthma symptoms in mice challenged with OVA. Then, we predicted the potential targets of nobiletin through pharmacophore model matching. It was found that the strongest target binding protein of nobiletin was PDE4B. Molecular docking, MD simulation, and SPR analysis further revealed a stable interaction between PDE4B and nobiletin. PDE4B enzyme inhibition experiments demonstrated that nobiletin exhibits inhibitory effects on PDE4B enzyme activity. Meanwhile, we observed activation of the cAMP-PKA-CREB pathway in the lung tissue. Accordingly, nobiletin treatment also reduced the level of inflammation in LPS-stimulated RAW264.7 cells and inhibited TGF-β1-induced ASM cell proliferation. Furthermore, it was confirmed that nobiletin could activate the cAMP-PKA-CREB pathway in RAW264.7 and ASM cells in vitro. Finally, siRNA tests provided further evidence for the dependence of PDE4B inhibition by nobiletin in exerting its anti-asthma effect (Figure 13).

Pro-inflammatory factors such as IL-5, IL-13, TNF-α, and IL-6 play a leading role in the pathogenesis of allergic asthma, including the growth of eosinophils, the isotypic transition of B cells to IgE production, chemotaxis of neutrophils, and bronchial contraction [51,52,53]. In the present study, nobiletin treatment effectively decreased OVA-IgE levels in the serum and the cytokines mentioned above. Hypereosinophilic syndrome is often present in patients with asthma. Therefore, a decrease in the number of eosinophils seems to be a logical therapeutic strategy for the treatment of allergic asthma [54]. Our study showed that nobiletin could reduce the number of inflammatory cells, including eosinophils, in the BALF. As shown in the pictures of the stained lung tissue sections, nobiletin treatment could also remarkably improve inflammatory cell infiltration and remarkably inhibit mucus hypersecretion induced by OVA in asthmatic mice. Furthermore, pulmonary function detection showed that nobiletin decreased Penh in mice, resulting in reduced airway hyperresponsiveness, which is a characteristic of asthma [55]. These results provide a comprehensive perspective for evaluating the therapeutic effects of nobiletin on allergic asthma.

Then, we further investigated the potential targets of nobiletin. It was virtually matched with pharmacophore models using the Ligand Profiler model in the Discovery Studio operating platform [56]. We found that nobiletin could interact with a variety of proteins, and the strongest interaction was with PDE4B. Molecular docking and MD tests further demonstrated a stable binding capacity between nobiletin and PDE4B. Then, SPR detection was performed to detect the affinity index of the two, which was 30.29 μM. In addition, the PDE enzyme assay directly demonstrated the inhibitory effect of nobiletin on PDE4B with an IC_50_ of 741.5 nM. The difference between the KD and IC_50_ values may be due to experimental errors. Inhibition of PDE4 could increase cAMP concentrations, which has been shown to be closely associated with inflammation [57]. Our results also support a role for cAMP in the inhibition of inflammation. Immunohistochemical analysis of mice lung tissue showed that cAMP levels increased after nobiletin intervention. Meanwhile, it was demonstrated that nobiletin treatment remarkably increased PKA-C levels and activated CREB phosphorylation, while also reducing the levels of p-NF-κB in the lung tissue of mice. As NF-κB has been shown to be critical in Th2 cell differentiation, inhibition of the NF-κB p65 nuclear entry response could also alleviate asthma symptoms. Our results indicated that nobiletin may activate the cAMP-PKA-CREB and NF-κB signaling pathways through the inhibition of PDE4B.

To further evaluate the anti-asthmatic activity of nobiletin in vitro, LPS was used to induce inflammation in RAW264.7 cells. Nobiletin remarkably reduced the levels of NO, TNF-α, and IL-6 in LPS-stimulated RAW264.7 cells. Several studies have reported that Citri Reticulatae Pericarpium has a therapeutic effect on allergic diseases and could reduce the levels of LPS-induced inflammation in RAW264.7 cells [58,59], which is consistent with the anti-inflammatory activity of nobiletin in our experiments. Moreover, nobiletin could activate the cAMP-PKA-CREB signaling pathway in RAW264.7 cells, which is consistent with the results observed in mice lung tissue. To determine the role of PDE4B in the increase in cAMP levels by nobiletin intervention, we investigated the changes in cAMP and PKA-C levels after nobiletin treatment in PDE4B siRNA-transfected cells. It was found that intracellular cAMP levels did not increase remarkably after nobiletin treatment compared to the group without nobiletin treatment, suggesting that the increase in cAMP levels induced by nobiletin may depend on PDE4B inhibition.

Airway remodeling caused by abnormal proliferation of ASM cells is an important mechanism in asthma progression [60,61]. In vitro investigations have shown that TGF-β1 can stimulate ASM cell proliferation and migration [62,63]. Our results also demonstrated that TGF-β1 can induce ASM cell proliferation and is accompanied by an increase in Smad2/3 phosphorylation levels, which is consistent with previous findings. Our study has shown for the first time that nobiletin suppressed TGF-β1-induced cell proliferation in ASM cells and supported the idea that nobiletin functions to inhibit airway remodeling in vivo [64]. Additionally, studies have shown that TGF-β1 can regulate intracellular cAMP levels through a mechanism dependent on Smad2/3, mediating physiological processes [65].The present study also confirmed that TGF-β1 could reduce the level of intracellular cAMP, and, more importantly, in the presence of nobiletin, the level of cAMP was increased, and the levels of p-Smad2/3 were reversed. cAMP activates downstream signal PKA, which is highly associated with smooth muscle relaxation of the airways and airway remodeling. Western blotting results also confirmed that the cAMP-PKA-CREB signaling pathway was activated after the intervention of nobiletin. Additional studies are needed to determine the interaction between TGF-β1 and cAMP signaling.

In conclusion, our study provides evidence for the activity of nobiletin in improving asthma symptoms by targeting PDE4B and subsequently activating the cAMP-PKA-CREB signaling pathway. Studies have shown that nobiletin exerts its activity through a multi-target synergistic pathway to achieve a better therapeutic effect. However, its extensive effects on targets also represent non-specific effects, which may result in certain toxic effects. Additionally, since the catalytic domain of PDE4 is highly conserved, nobiletin may also inhibit PDE4D and cause nausea and vomiting. Further studies are needed to determine the balance between the safety and efficacy of nobiletin. Even so, the results of several in vivo and in vitro experiments demonstrated that nobiletin should be considered a potential candidate for the treatment of asthma. The target of nobiletin and the signaling pathway identified in this study are worthy of further investigation.

## 4. Materials and Methods

### 4.1. Drugs and Reagents

Nobiletin (purity > 98%) was purchased from Nanjing Spring & Autumn Biological Co., Ltd. (Nanjing, China). Doxofylline was purchased from Shanghai Aladdin Reagent Co., Ltd. (Shanghai, China). Apremilast was purchased from Solarbio Biotechnology Co., Ltd. (Beijing, China). Aluminum hydroxide (Al(OH)_3_) was purchased from Fenghui Biotechnology Co., Ltd. (Shanghai, China). OVA was purchased from Sigma Biology Co., Ltd. (St. Louis, MO, USA). Mouse TNF-α, IL-6, IL-5, IL-13, OVA-IgE, and cAMP ELISA kits were purchased from Shanghai Fankewei Biotech Co., Ltd. (Shanghai, China). The BCA protein quantification kit, nitric oxide detection kit, and SDS-PAGE rapid gel preparation kit were purchased from Shanghai Beyotime Biotechnology Co., Ltd. (Shanghai, China). The antibody against PDE4B (ab170939) was purchased from Abcam (Cambridge, UK). Antibodies against p-NF-κB (#3033), NF-κB (#8242), p-CREB (#9198), CREB (#9197), p-Smad2/3 (#8828), Smad2/3 (#8685), and PKA-C (#5842) were purchased from Cell Signaling Technology (CST) (Danvers, MA, USA). Fetal bovine serum (FBS) was purchased from Excell (Shanghai, China), and DMEM was purchased from Wuhan Servicebio Technology Co., Ltd. (Wuhan, China). The mouse anti-cAMP antibody (sc73761) was purchased from Santa Cruz Biotechnology Inc. (Dallas, TX, USA). RNAFit and siRNA were purchased from HANBI Biotechnology Co., Ltd. (Shanghai, China). His-tagged PDE4B was obtained from Bioesn Biotechnology Co., Ltd. (Beijing, China). Tris buffer was purchased from Beyotime Biotechnology Co., Ltd. (Shanghai, China). cAMP was obtained from Solarbio Biotechnology Co., Ltd. (Beijing, China). Calf Intestinal Alkaline Phosphatase (CIAP) was purchased from Yeasen Biotechnology Co., Ltd. (Shanghai, China). PDE4B protein was obtained from SignalChem Biotech Inc. (Vancouver, BC, Canada). Ammonium molybdate was purchased from Shanghai Macklin Biochemical Technology Co., Ltd. (Shanghai, China). Malachite green was obtained from Sangon Biotech Co., Ltd. (Shanghai, China). HClO_4_ was obtained from Sinopharm Chemical Reagent Co., Ltd. (Shanghai, China).

### 4.2. Animals

Female BALB/c mice (6 to 8 weeks old) were obtained from Jinan Pengyue Laboratory Animal Co., Ltd. (Jinan, China). The animals were housed in polypropylene cages and kept under standard conditions of 12 h of alternating light and dark cycles at a constant temperature of 22 °C and a relative humidity of 50%. All procedures were approved by the Animal Experiments Ethics Committee of the Ocean University of China (OUC-SMP-2021-02-02).

### 4.3. Animal Experimental Protocol

In total, 100 mice were randomly divided into five groups, with 20 mice in each group as follows: the control group, ovalbumin (OVA) group, OVA + nobiletin group (7.5 mg/kg), OVA + nobiletin group (15 mg/kg), and OVA + doxofylline group (10 mg/kg). OVA, the main protein in egg white, is a common asthma inducer that can be used to construct a mouse model of asthma. Briefly, on days 0, 7, and 14, each mouse was sensitized by intraperitoneal injection of 20 µg OVA and 2 mg Al(OH)_3_ in 200 µL PBS; 200 µL PBS was injected intraperitoneally in the control group. On days 21, 22, and 23, OVA-challenged mice were exposed to 5% OVA ultrasonic atomization for 30 min each day. The mice in the control group were challenged with an equal amount of PBS. From day 17 to day 23, the mice were treated with nobiletin (7.5 mg/kg, 15 mg/kg) or doxofylline (10 mg/kg) by intraperitoneal injection once a day. The dosage selection for nobiletin was based on research on eupatilin in asthmatic mice, which is also a flavonoid compound. We found that nobiletin was completely soluble at 15 mg/kg, so 15 mg/kg, as well as 7.5 mg/kg reduced by a factor of two, was chosen as the high and low doses for the study. This method is consistent with previous research on eupatilin. Nobiletin and doxofylline were dissolved in normal saline containing 3% DMSO to prepare a homogeneous solution, and the control and model groups were injected with normal saline containing the same concentration of DMSO. The mice were euthanized on day 24 and sampled for testing.

### 4.4. BALF Collection and Leukocyte Counts

The mice were anesthetized by injection of 1% sodium pentobarbital. The trachea was intubated, and the lungs were washed twice with 0.7 mL of PBS to collect the BALF. Then, the BALF was centrifuged at 827× *g* for 5 min. The supernatant was absorbed. The total number of white blood cells, neutrophils, lymphocytes, monocytes, and eosinophils in the BALF was detected by the ProCyte Dx^®^ hematology analyzer (IDEXX Laboratories Inc., Westbrook, ME, USA).

### 4.5. Determination of IL-5, IL-13, IL-6, and OVA-IgE Levels

The levels of IL-5, IL-13, and IL-6 in the BALF supernatant and OVA-IgE in the serum were detected using a double sandwich ELISA, as described in the corresponding kit instructions.

### 4.6. Detection of Airway Hyperresponsiveness

Airway hyperresponsiveness in mice was detected in asthmatic animals using whole-body plethysmography (WBP-4MR, TOW, Shanghai, China) [66]. The naturally active mice were placed in the instrument and atomized with different concentrations of acetylcholine (0, 6.25, 12.5, 25, and 50 mg/mL). The Penh of the mice was recorded.

### 4.7. Histopathologic Evaluation of the Lung Tissue

The lung tissues of the mice were fixed with 4% paraformaldehyde, dehydrated, and embedded in paraffin before being sliced and stained with H&E and PAS. The lung histopathological changes and mucus content were observed under a light microscope. Three fields were randomly selected and photographed at 400× magnification.

### 4.8. Ligand Profiling and Molecular Docking

Compound profiling was performed using Discovery Studio software version 2021 (BIOVIA, Paris, France). Ligand Profiler, based on PharmaDB, was used to transform the 2D structure of nobiletin into a 3D structure in Discovery Studio [67]. Nobiletin was then connected to each protein in the database, and it was automatically matched with multiple pharmacophore models, scored, and ranked [68].

All protein structure files of PDE4B were downloaded from RCSB PDB (https://www.rcsb.org/, accessed on 13 December 2023). The protein structures were prepared in the Minimize Structure module with the ff14SB force field on UCSF Chimera (http://www.cgl.ucsf.edu/chimera/, accessed on 13 December 2023). Finally, all the structures were converted to pdbqt format with all hydrogens kept by the rdkit2pdbqt.py script (https://github.com/biocheming/watvina, accessed on 7 December 2023). The docking box size of each protein is 20 Å × 20 Å × 20 Å. Docking studies of PDE4B structures and nobiletin were performed using Watvina (https://github.com/biocheming/watvina, accessed on 13 December 2023), which was developed by our group. Based on the Autodock Vina molecular docking engine, Watvina is optimized for the scoring function and conformation searching algorithm. The scoring function of Watvina consists of Van der Waals, hydrogen bonds, polar-polar repulsion, and hydrophobic attraction. Unlike Autodock Vina, Watvina considers the contribution of all hydrogen atoms. Conformation searching adopts a simplified genetic algorithm BFGS combination strategy; in addition, a torsion penalty is calculated for conjugated rotatable single bonds.

### 4.9. MD Simulation

MD simulations were performed using the OpenMM software version 7.7.0 (https://openmm.org/, accessed on 28 August 2024, Stanford University, Palo Alto, CA, USA and Columbia University, New York, NY, USA), which played a significant role in inspecting ligand-binding interactions and stability. The complex solution system was generated on CHARMM-GUI (http://www.charmm-gui.org, accessed on 13 December 2023), an online website for preparing complex biomolecular systems for molecular simulations. The molecular system was processed using the ff19SB and gaff2 force fields, solvated with crystallographic water (TIP4P-EW) molecules under rectangular periodic boundary conditions with a 10 Å buffer region, and neutralized by adding Na+ as counterions. A 125 ps equilibrium MD simulation was performed with the NVT ensemble, which adopted the L-BFGS algorithm with 5000 steps and a harmonic restraining force imposed on the backbone (force constant 400 kJ/mol/nm^2^) and sidechains (force constant 40 kJ/mol/nm^2^) to run a local energy minimization. Then, a production MD simulation was carried out by adding the MonteCarloBarostat to control the pressure at 1 bar and a temperature of 303.15 K for 100 ns. A time step of 4 fs was set, and the energy and structures were recorded every 100 ps. The results of the MD simulation were visualized and analyzed using VMD software version 1.9.4 (http://www.ks.uiuc.edu/Research/vmd//, accessed on 28 August 2024, University of Illinois at Urbana-Champaign, Urbana, IL, USA).

### 4.10. SPR

To validate the binding capacity of nobiletin and PDE4B protein, an SPR test was used to inject 0.5 mM NiCl_2_ flow buffer into the activated surface under an injection pulse of 350 mM EDTA for 1 min. His-tagged PDE4B was then injected into a flow buffer and bound to the surface of the NTA chip. Different concentrations of nobiletin (7.8125, 15.625, 31.25, 62.5, 125, and 250 μM) were prepared in HBS-EP buffer solution. The Biacore T200 evaluation software version 3.1 (Cytiva, Washington, DC, USA) was used to analyze and fit the experimental data. The binding affinity constants of nobiletin and PDE4B protein were calculated.

### 4.11. PDE4B Inhibition Assay

Briefly, the blank, control, and nobiletin/apremilast groups were set up in a 96-well plate. The following reagents were added to each well in turn: 83.3 μL of Tris buffer, 16.2 μL of 0.5 mM cAMP, 12 μL of CIAP, and 13.5 μL of different concentrations of nobiletin/apremilast (prepared with Tris buffer). CIAP is an enzyme that can hydrolyze the 5′-terminal phosphate group of cAMP. An equal volume of Tris buffer was added to the blank and control groups. Finally, 10 μL of PDE4B protein was added, and the same volume of Tris buffer was added to the blank group. The mixture was incubated at 37 °C for 20–60 min. Then, 25 μL of 40% HClO_4_ was added, followed by 30 μL of 280 mM ammonium molybdate and 30 μL of 1 mM malachite green. After incubation at 37 °C for 15 min, the absorbance was measured at 630 nm [69]. The formula for calculating the inhibition rate is as follows: A_0_ represents the absorbance of the blank group, A_1_ represents the absorbance of the control group, and A_2_ represents the absorbance of the nobiletin/apremilast group.

$$\mathrm{Inhibition}\;\mathrm{rate}\;\left(\%\right)=\frac{\left(A_{1}-A_{0})-(A_{2}-A_{0}\right)}{\left(A_{1}-A_{0}\right)}\times 100\%$$

### 4.12. Immunohistochemical Staining

cAMP expression was observed in mouse lung tissue by immunohistochemistry. Briefly, tissue sections were pretreated in a microwave oven (80–90 °C, 600–700 W, and 10 min) to inactivate the enzymes, blocked, and incubated with primary and secondary antibodies. The samples were chromogenized, counterstained, decolorized, sealed, and scanned using a digital pathology slide scanner.

### 4.13. Cell Culture and Treatments

The RAW264.7 cell line was obtained from the Chinese Academy of Sciences cell bank and cultured in a 5%CO_2_ incubator at 37 °C with 10% FBS added to DMEM. All experiments were performed using cells between the third and sixth passages. ASM cells were purchased from Fu Heng Biotechnology Co., Ltd. (Shanghai, China) and cultured in a 5%CO_2_ incubator at 37 °C with 10% FBS added to DMEM. All experiments were performed using cells between the third and sixth passages. Nobiletin was dissolved in DMSO (0, 3.75, 7.5, 15, 30, 60 μmol/L). The control group was also treated with the same volume of vehicle (containing the same concentration of DMSO).

### 4.14. MTT Assay

RAW264.7 cells were placed in 96-well plates at 3 × 10^4^ cells/well for 12 h and treated with different concentrations of nobiletin (0, 3.75, 7.5, 15, 30, and 60 μmol/L) for 24 h. After that, 20 μL of 5 mg/mL MTT solution was added to the cells and incubated for 4 h to form purple crystals. The crystals were dissolved by replacing the culture medium with 150 µL of DMSO. Finally, the absorbance was measured at 570 nm.

ASM cells were placed in 96-well plates at a density of 1 × 10^4^ cells/well for 12 h and were treated with different concentrations of nobiletin (0, 3.75, 7.5, 15, and 30 µmol/L) for 24 h. MTT was added to measure the absorbance.

### 4.15. Measurement of NO, TNF-α, and IL-6

RAW264.7 cells were seeded in 96-well plates at 3 × 10^4^ cells/well for 12 h, then treated with 1 mg/mL of LPS and nobiletin at the same concentration as before for 24 h. The nitrite content was determined as previously described [70]. 50 μL of cell supernatant was mixed with Griess I and Griess II. The absorbance was measured at 540 nm. The contents of TNF-α and IL-6 in the cell supernatant were detected using the double antibody sandwich ELISA technique.

### 4.16. Immunofluorescence Analysis

RAW264.7 cells were seeded at 1 × 10^5^ cells/well for 12 h and treated with 1 mg/mL of LPS and nobiletin for 24 h. The cells were fixed with 4% paraformaldehyde for 20 min at 4 °C and blocked with 5% BSA for 1 h. Then, they were incubated with an NF-κB antibody overnight. Subsequently, the secondary antibody was incubated at 37 °C for 1 h in the dark, followed by incubation with DAPI for 10 min in the dark. Finally, fluorescence was measured under a fluorescence microscope (Nikon Corporation, Tokyo, Japan). Images were analyzed using ImageJ software version 1.53a (NIH, Bethesda, MD, USA). NF-κB fluorescence, represented by FITC, was used as the total fluorescence, while NF-κB overlapping with DAPI was used as the nuclear NF-κB. The ratio of nuclear NF-κB to total NF-κB was calculated as the nuclear entry rate.

### 4.17. Cell Proliferation Assay

ASM cells were seeded in 96-well plates at a density of 1 × 10^4^ cells/well for 12 h and then starved in serum-free medium for 12 h before treatment with 10 ng/mL TGF-β1 and different concentrations of nobiletin (0, 3.75, 7.5, 15, and 30 µmol/L) for 24 h. Cell proliferation was detected using the MTT assay. The absorbance was measured at 570 nm.

### 4.18. Cell Migration Assay

ASM cells were seeded in 6-well plates at a density of 6 × 10^5^ cells/well for 12 h. Three lines were randomly drawn vertically in each well. Cell fragments were cleared with PBS washes three times, and cultures were changed to serum-free medium for 12 h before treating the cells with 10 ng/mL TGF-β1 and nobiletin. Cells were observed and photographed at 0, 12, 24, and 48 h after treatment.

### 4.19. Measurement of cAMP Levels

The cells were seeded at a density of 8 × 10^5^ cells/well for 12 h. They were then treated with TGF-β1 and different concentrations of nobiletin (0, 3.75, 7.5, 15, and 30 μmol/L). After treatment, the cells were collected, broken by repeated freezing and thawing, and then centrifuged. The cAMP content was detected using ELISA, a double antibody sandwich method.

### 4.20. siRNA Transfection

RAW264.7 cells were seeded in a 6-well plate until they reached about 30–50% confluency. DMEM medium, RNAFit reagent, and siRNA were combined in a microcentrifuge tube according to the manufacturer’s protocol and incubated for 10 min. The growth medium was then replaced with a new complete medium. The siRNA mixture was added to the cell culture plates according to the experimental design and incubated. Cells were collected 72 h after transfection. The following list contains the target sequences of these siRNAs: mmu-PDE4B-si1 (positive-sense strand: CGUUCUUAGACAAGCAGAATT; antisense strand: UUCUGCUUGUCUAAGAACGTT), mmu-PDE4B-si2 (positive-sense strand: GAUUCAAAUUGCUACAAGATT; antisense strand: UCUUGUAGCAAUUUGAAUCTT), mmu-PDE4B-si3 (positive-sense strand: GGUUCAACCGGAUGCUCAATT; antisense strand: UUGAGCAUCCGGUUGAACCTT), NT siRNA (positive-sense strand: UUCUCCGAACGUGUCACGUTT; antisense strand: ACGUGACACGUUCGGAGAATT)

### 4.21. Western Blotting Analysis

As described in a previous study [71]. Briefly, lung tissue and cell samples were lysed using cell lysis buffer. The samples were collected and centrifuged at 13,225× *g* for 10 min. The supernatant was denatured at 100 °C for 10 min. A 6–10% separation gel/5% concentrated gel was prepared, and the proteins were then transferred to an NC membrane. The membrane was blocked with 5% BSA for 2 h, exposed to the corresponding primary antibody, and incubated overnight at 4 °C. The membranes were washed with 1 × TBST three times for 5 min each time. The second antibody was incubated at room temperature for 60 min and the membranes were washed with 1 × TBST three times, for 5 min each time. Finally, the strips were visualized using an alkaline phosphatase color development kit. ImageJ software version 1.53a. (NIH, Bethesda, MD, USA) was used for gray integration analysis.

### 4.22. Statistical Analysis

The data in this study are expressed as the mean ± SEM. Differences among groups were analyzed using one-way ANOVA with Tukey’s multiple comparisons (GraphPad Prism version 8.0.2, GraphPad Software, San Diego, CA, USA). All groups were compared with the control or model group to obtain *p* values. *p* < 0.05 was considered statistically significant.

## Data Availability

The original contributions presented in the study are included in the article material, further inquiries can be directed to the corresponding authors.

## Abbreviations

MTT	3-(4:5-dimethylthiazol-2-yl)-2,5-diphenyl tetrazolium bromide
FBS	fetal bovine serum
BALF	bronchoalveolar lavage fluid
OVA	ovalbumin
LPS	lipopolysaccharide
TGF-β1	transforming growth factor β1
SPR	surface plasmonic resonance
ASM	airway smooth muscle
PDE4B	phosphodiesterase 4B
MD	molecular dynamics.
Penh	enhanced pause
cAMP	3′,5′-cyclic adenosine monophosphate
PKA	protein kinase A
CREB	cyclic-AMP response binding protein
